# Stress, Anxiety and Depression among a Cohort of Health Sciences Undergraduate Students: The Prevalence and Risk Factors

**DOI:** 10.3390/ijerph18063269

**Published:** 2021-03-22

**Authors:** Muhammad Faris Fauzi, Tengku Shahrul Anuar, Lay Kek Teh, Wai Feng Lim, Richard Johari James, Rohana Ahmad, Mawarni Mohamed, Sahol Hamid Abu Bakar, Farida Zuraina Mohd Yusof, Mohd Zaki Salleh

**Affiliations:** 1 Integrative Pharmacogenomics Institute, Universiti Teknologi MARA, Cawangan Selangor, Kampus Puncak Alam 42300, Selangor, Malaysia; faris.ipromise@gmail.com (M.F.F.); tengku9235@uitm.edu.my (T.S.A.); tehlaykek@uitm.edu.my (L.K.T.); limwaifeng@uitm.edu.my (W.F.L.); richard@uitm.edu.my (R.J.J.); drrohana@uitm.edu.my (R.A.); fzuraina@uitm.edu.my (F.Z.M.Y.); 2 Faculty of Applied Sciences, Universiti Teknologi MARA, Shah Alam 42300, Selangor, Malaysia; 3 Centre of Medical Laboratory Technology, Faculty of Health Sciences, Universiti Teknologi MARA, Cawangan Selangor, Kampus Puncak Alam 42300, Selangor, Malaysia; 4 Faculty of Pharmacy, Universiti Teknologi MARA, Cawangan Selangor, Kampus Puncak Alam 42300, Selangor, Malaysia; 5 Faculty of Dentistry, Universiti Teknologi MARA, Cawangan Selangor, Kampus Sungai Buloh 47000, Selangor, Malaysia; 6 Faculty of Education, Universiti Teknologi MARA, Cawangan Selangor, Kampus Puncak Alam 42300, Selangor, Malaysia; mawarnim@uitm.edu.my; 7 Faculty of Civil Engineering, Universiti Teknologi MARA, Shah Alam 40450, Selangor, Malaysia; SH.AbuBakar@insead.edu

**Keywords:** mental health, psychological, risk factors, student health, Malaysia

## Abstract

Stress, anxiety, and depression (SAD) have a negative impact on the learning and academic performance of university students. Hence, this study aimed to determine the prevalence, as well as the risk factors associated with SAD among a cohort of students pursuing undergraduate degree courses in health sciences. This is part of the strategy in building a healthy nation. A questionnaire containing socio-demographic factors and the short version of Depression, Anxiety, and Stress Scale-21 (DASS-21) was used to assess the likelihood of psychological distress. Logistic regression analysis was conducted to determine the risk factors of SAD. In total, 449 students completed the questionnaire (93.9% response rate). Of these, 65% had stress, 85.1% had anxiety and 51.4% had depression. Most cases of stress (74.6%) and depression (66.2%) were of normal-to-mild level, while 74.6% of them showed moderate-to-extremely severe anxiety. There was a statistically significant association between stress score and the year of study. In the regression analysis, poor sleep quality and fatigue were risk factors of anxiety and depression, whereas low-grade fever and frequent headaches were risk factors for stress and anxiety. Stress, anxiety, and depression scores were significantly higher among students studying medical imaging. A substantial proportion of health science students are suffering from SAD. This study recommends screening and close monitoring of the above-mentioned predictors and the formulation of comprehensive intervention strategies for students with SAD.

## 1. Introduction

In recent years, the growing competitive nature of higher education has led to an exacerbation of common academic stressors among university students, some of whom end up suffering from mental health issues. Based on the literature, undergraduate students in the medical or health science streams were more likely to be afflicted with psychological distress such as anxiety, depression, and feeling suicidal [1,2]. It is well known that healthcare education has very high complexity and uniqueness whereby students need to achieve academic excellence, clinical competencies, and good interpersonal skills. As such, it aims to produce well-trained frontline health workers who are capable of taking the responsibility of advancing public health and achieving high levels of patient-centered care. This requires many years of stressful studying and persistent training. On top of classroom learning, they also need to undergo an extensive evaluation that involves both theoretical and practical assessments [3]. Such a complex learning environment and the constant struggle in the training to become qualified healthcare providers can lead to psychological distress. Studies have shown that the mental and emotional well-being of the students can be indirectly affected by certain components of the training [4]. All these negative implications must be given serious consideration because they can be a potential threat to patient care and safety.

Based on many published studies, the performance of university students can be affected by mental health disorders such as stress, anxiety, and depression (SAD) that are common among the students. These factors have been associated with a lower quality of life and the development of many life-threatening diseases such as cardiovascular diseases and cancers [5,6]. Recent meta-analyses reported that Asian university students in medicine and nursing courses often experienced a high prevalence of depression at 11% and 43%, respectively [7,8]. Mojs et al. [9] investigated mental health disorders among 1183 Polish health sciences students and reported an overall depression rate of 6.5% among physical therapy students. In Australia and the United Kingdom, a study conducted among 434 physical therapy students found that the main type of stress experienced by students was academic-related stress, in which 71% of the students perceived their studies as more difficult than expected [10]. Another research conducted in the United States showed that occupational therapy students were also susceptible to school-related stress. About two-thirds (66.4%) of the respondents were overwhelmed and confused about their study expectations [11]. Furthermore, Jacob et al. [12] performed a study in the Middle East and showed significant levels of stress among 312 healthcare students in the courses of physical therapy, communication disorders, and nutrition. However, there was no significant relationship between stress and demographic variables.

In general, many studies had demonstrated the association between risk factors of SAD with academic performance among university students. The risk factors include the pressure to succeed and uncertainty of their future after graduation [13,14]. These factors are especially common among healthcare students, and female students are more likely to be affected. The stressors that affect the healthcare students can either be exogenous (associated with the study and training load) or endogenous (related to personality traits) [15]. There are many stressors for students majoring in healthcare courses such as medicine, dentistry, nursing, and physical therapy. These stressors include academic stressors (difficulties in understanding and learning a new syllabus, high workload, frequency of tests), psychosocial stressors (high paternal expectations, and financial strain), and socio-demographic stressors (gender, marital status, the levels of parents’ education, and cultural background) [16,17]. These stressors can cast a negative impact on the academic performance, social relationships, future employment, substance abuse, and marital life of the students, and subsequently, lead to substance abuse [18]. A one-year prospective study demonstrated a significant escalation in the prevalence of SAD when students advanced to clinical training years and resulted in undesirable outcomes including suicide [19].

Malaysia is not spared from the global issue of poor mental health among university students. As one of the leading countries that provide higher education in the Asia-Pacific region [20], the number of Malaysian students with mental health disorders has increased from 10% in 2011 to 20% in 2016 [21]. A recent publication highlighted that youths between 16 to 24-years old recorded the highest prevalence of psychological distress as a result of academic-related pressure [22]. Timely detection and management of mental health disorders among young adults in schools and universities are essential to safeguard public health. To the best of our knowledge, most of the published studies in this area targeted general undergraduate students. There is very little research that specifically investigated the risk factors or the stressors of mental health disorders among health science students in Malaysia. This is a vital area of research because the psychological wellbeing and mental health of the students are necessary to ensure that they can achieve the intended growth and development in life. Therefore, this study aimed to determine the prevalence and the risk factors associated with SAD among the health science students of different academic majors at a local university in Malaysia.

## 2. Materials and Methods

### 2.1. Study Design, Setting, and Participants

A cross-sectional study was conducted to recruit students at the Faculty of Health Sciences at Universiti Teknologi MARA (UiTM), Puncak Alam, Malaysia. The majority of the students in this institution are Bumiputera ethnic groups. 

The study participants were students in the first, second, third, and fourth years of three academic courses (Medical Laboratory Sciences, Medical Imaging, and Nursing). All these courses run for four years. Part-time students and students who did not register in the respective semesters were excluded.

### 2.2. Sample Size

The sample size was calculated using OpenEpi (www.openepi.com) (accessed on 4 March 2021), an open-source statistical software. The prevalence of anxiety used for the calculation was 34% as reported by Shamsuddin et al. [17], the significance level was set at 0.05, and there was a 40% of non-response rate and minimum sample size of 432.

### 2.3. Data Collection

Participants recruitment started in March and ended in June 2018. Flyers were distributed to students in the Medical Laboratory Sciences, Medical Imaging, and Nursing programs. The students who consented to participate in the study were gathered in the lecture halls. Study protocols and objectives were explained to the participants before they signed the informed consent form. Self-administered sociodemographic and DASS-21 questionnaires were distributed to the students to be filled up within 20 min. The researchers stayed with the participants throughout the data collection to answer queries from the participants. The filled questionnaires were anonymous and were collected by the researchers. This was done in week 10 of the first term because it was the most suitable time without any examination. This was deemed the most suitable time for recruitment as the participants had completed all the assignments. Students under psychiatric treatment were excluded. No incentive was given for participation. 

### 2.4. Questionnaire

A two-section questionnaire was used in this study. The first section includes information on the socio-demographic data (gender, age, and marital status), academic-related data (course major, current semester, university CGPA, and education fund), health and lifestyle (self-evaluation of health and diet, exercise habits, smoking, and weight loss), medical history (presence of physical and mental dysfunctions), food diary (dietary pattern of selected food items and frequency of consumption, use of drugs, and herbs supplements). 

In the second section, the Depression Anxiety Stress Scale-21 (DASS 21) was applied. It is a 21-item self-report tool measuring attitudes and symptoms of SAD. There are 21 items with 7 items each for SAD [23]. DASS-21 is a simplified version of the original DASS-42 which can measure all the psychometric properties to determine the state of SAD [24]. For stress, the subscale focuses on persistent arousal and tension. As for anxiety, the items assess fear response and psychological arousal, whereas depression includes low mood, low self-esteem, and a poor outlook for the future [25]. Participants answered on a four-point Likert scale (0 = Did not apply to me at all, 1 = Applied to me to some degree, or some of the time, 2 = Applied to me to a considerable degree, or a good part of the time, and 3 = Applied to me very much or most of the time). Based on the DASS-42 manual, scores from each subscale were summed up and then multiplied by two to allow its interpretation. Each subscale score ranges between 0 and 42 in which higher scores indicate greater levels of distress [23]. The levels of SAD were categorized as “normal”, “mild”, “moderate”, “severe”, and “extremely severe” [23] which is shown in Table 1. For this study, a modified Lovibond scoring scale was used in data analysis. The scores of SAD were categorized as dichotomous responses of normal or abnormal. The cut-off scores of ≤9 in depression, ≤7 in anxiety, and ≤14 in stress were considered normal and a score of ≥10 in depression, ≥8 in anxiety, and ≥15 in stress were considered abnormal [26].

It is important to take note that DASS-21 only reflects the severity of the symptoms, but it is not a diagnostic tool. This screening tool is widely accepted and applied due to its reliability, user-friendliness, and ease of administering to the general population. Furthermore, no special training is needed to administer the instrument [17]. Based on the normative sample in a recent study, DASS-21 showed good reliability based on its Cronbach’s alpha value, i.e., depression scale (α = 0.91), anxiety scale (α = 0.84), and stress scale (α = 0.90) [27]. Furthermore, the translated versions of DASS-21 are available in several languages and they have been validated in various populations, including Malaysia [28].

The study questionnaire was developed via discussion with an expert panel before being validated in a pilot study consisting of 30 students who were not included in the data collection and analysis of the main study. The age of these 30 participants ranged between 18 and 23 years old with a mean age of 19.6 years (SD ± 0.967). They reported that the questionnaire was clear and easy to follow. From the pilot study, the DASS-21 showed high internal consistency as the Cronbach’s alpha coefficients were 0.83 for the depression scale, 0.70 for the anxiety scale, and 0.77 for the stress scale. In short, the study survey was statistically sound and appeared clear to the participants. 

### 2.5. Data Analyses

Data analyses were carried out using SPSS software for Windows (version 23.0, IBM Corporation, Armonk, NY, USA). Data cleaning was done to detect missing values, coding errors, or any illogical data values. Continuous data were reported as means and standard deviations, whereas categorical data were reported as frequencies and percentages. Bivariate analyses between categorical variables were run using the chi-square test. In multivariable analysis, binary logistic regression was performed. In binary logistic regression, the first simple logistic regression (SLR) was performed. The dependent variables were analyzed as a dichotomy: 0 = normal cases in which the participants do not experience SAD, while 1 = abnormal cases where participants experience SAD. Independent variables with *p* < 0.25 were included in the multiple logistic regression (MLR). Then, MLR was performed using the Enter method. Odds ratio (OR) and 95% confidence interval (CI) were calculated for the risk factors of SAD. *p* < 0.05 was considered statistically significant. The goodness-of-fit showed the logistic regression model fit the data well with *p* = 0.752 (depression model), *p* = 0.150 (anxiety model), and *p* = 0.105 (stress model). To investigate the possible association between students’ CGPA and SAD scores, Pearson correlations were performed with *p* < 0.05 considered statistically significant.

### 2.6. Ethical Approval and Consent to Participate

The study was conducted based on the principles of the Declaration of Helsinki. Ethical approval for this study was granted by the Research Ethics Committee of Universiti Teknologi MARA (REC/365/16). Students with higher psychological distress scores were given appropriate referrals for counseling. 

## 3. Results

### 3.1. Socio-Demographic Characteristics of the Participants

Of the 478-health science undergraduate invited to participate in the study, 449 students agreed to participate and completely filled out the questionnaire, which is a response rate of 93.9%. Students who did not participate did not fulfill the inclusion criteria, were absent from class on data collection day, or refused to participate in the study. The sample consisted of 90.1% females (female to male ratio = 5.5:1). Participants’ ages ranged between 19 and 28 years, with a mean age of 21.86 (±1.76). Participants were from the first- through fourth study year. The mean CGPA of the participants was 3.32 out of 4.00. The majority of the participants were Malays (95.5%). More than half of the participants reported that they received scholarships throughout their study. Responses for monthly family income varied widely between the participants. The sample numbers for each major were 227 (50.6%) Medical Laboratory Sciences, 104 (23.2%) Medical Imaging, and 118 (26.3%) Nursing. Table 2 illustrates participants’ demographics and relevant characteristics.

### 3.2. Overall Severity of Depression, Anxiety, and Stress

**Figure 1:**
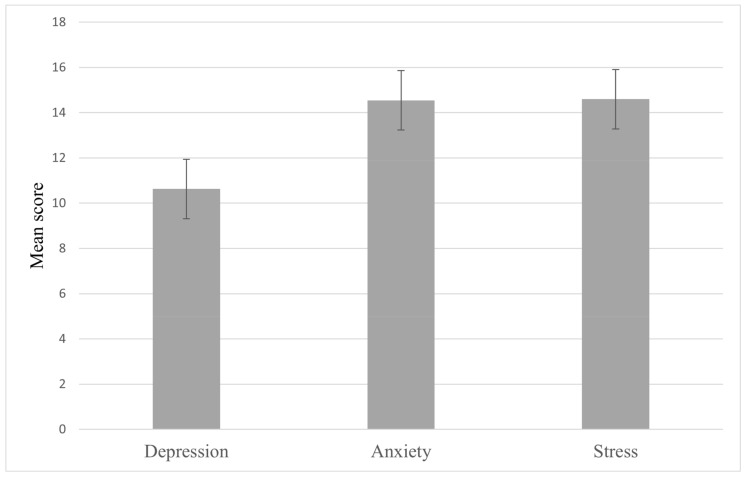
Means and standard deviations of the participants’ DASS-21 scale scores.

Participants’ DASS-21 mean scores were 14.60 (±7.06) for stress, 14.54 (±7.46) for anxiety, and 10.63 (±7.48) for depression (Figure 1). Table 3 shows the severity levels of SAD symptoms as measured by DASS-21 scale scores according to gender. Of the studied psychological distress types, anxiety was the most prevalent among participants with 85.1%, followed by stress (65%) and 51.5% of the participants suffered from depression. Most cases of stress (74.6%) and depression (66.2%) were normal-to-mild levels, while 74.6% of the students had moderate-to-extremely severe anxiety. Males and females did not differ significantly in the severity levels of stress (*p* = 0.645), anxiety (*p* = 0.119) and depression (*p* = 0.329). Our analysis further showed that anxiety was correlated with depression (r = 0.574, *p* = 0.000), depression was correlated with stress (r = 0.646, *p* = 0.000) and anxiety was correlated with stress (r = 0.684, *p* = 0.000). 

### 3.3. Factors Associated with Depression, Anxiety, and Stress Symptoms

Table 4 and Table 5 shows the multivariate analysis of factors associated with SAD. Fatigue was significantly associated with anxiety (OR = 0.854; 95% CI 1.427, 3.867; *p* = 0.001) and depression (OR = 0.506; 95% CI 1.044, 2.638; *p* = 0.032) symptoms. Interestingly, poor sleep quality also significantly associated with anxiety (OR = 0.633; 95% CI 1.003, 3.534; *p* = 0.049) and depression (OR = 0.795; 95% CI 1.377, 3.558; *p* = 0.001) symptoms. On the other hand, having low-grade fever was significantly associated with stress (OR = 0.597; 95% CI 1.042, 3.164; *p* = 0.035) and anxiety (OR = 1.072; 95% CI 1.242, 6.869; p = 0.014). Frequent headaches were also associated with stress (OR = 0.497; 95% CI 1.023, 2.640; *p* = 0.040) and anxiety (OR = 0.853; 95% CI 1.435, 3.839; *p* = 0.001) symptoms. Student from different academic majors reported significantly different stress, anxiety, and depression scores. Medical Imaging students were associated with stress (OR = 0.777; 95% CI 1.106, 4.278; *p* = 0.024), anxiety (OR = 0.821; 95% CI 1.053, 4.098; *p* = 0.036) and depression (OR = 1.365; 95% CI 2.046, 7.361; *p* = 0.001) symptoms compared to students from other academic majors (Medical Laboratory Sciences and Nursing). No significant correlation was observed between CGPA scores with stress (r = 0.068, *p* = 0.166), anxiety (r = 0.053, *p* = 0.283), and depression (r = −0.45, *p* = 0.365). Stress score was significantly associated with the year of study (*p* = 0.033) with higher stress scores in students of advanced year of studies.

## 4. Discussion

Undergraduate learning is an important development phase in the life of the students which is sensitive and challenging. It has been associated with high rates of psychological distress for them [29]. The present research addresses the gap in the literature on SAD among Malaysian university students by examining the socio-demographic factors and stressors. This study reported that SAD are commonly present (65%, 85.1%, and 51.4%, respectively) across students from all four academic years. Even though DASS-21 is not a diagnostic tool, symptoms of depression, anxiety, or stress of moderate or higher level of severity indicate a need for preventive strategies from the healthcare professionals and university administrative personnel. In a local study, Fuad et al. [30] also reported a high prevalence of anxiety (76%), depression (60%), and stress (47%) among medical students. These results are in line with study findings from other developing countries [3,31,32]. On the contrary, similar studies from developed countries such as the United States, United Kingdom, and Sweden reported lower levels of mental health symptoms among undergraduates [33,34,35]. In Malaysia, students are likely to experience stress from their studies in high schools and universities, as they set a high level of academic and performance. Moreover, the transition from high schools to universities can be challenging with unfamiliar education environments and lifestyle changes. Compared to their counterparts in private universities, public university students are expected to work harder and be more competitive, thereby imposing further stress on them [16]. Besides, the discrepancies in the prevalence of mental health disorders in different studies could be attributed to the application of different measurement tools. It could also be due to the variation in the understanding of self-confidence, self-evaluation, and adaptive behavioral styles between different societies [36]. 

In this study, we cannot conclude that gender plays a role in SAD due to the small sample size of male participants. Moreover, previous studies in Malaysia [37], Bangladesh [38], and India [39] also reported a lack of significant association between psychological distress and the gender of students. However, Dyrbye et al. [34], as well as Bayram and Bilgel [40], reported higher levels of SAD among females compared to males. Another study highlighted that females were twice as likely to be affected by anxiety disorders. What has been observed could be explained by another study that the underlying hormonal and biochemical differences between men and women could influence mental health [41]. In addition, the results of this study showed that the students’ academic year was significantly associated with stress. Similar findings were reported in studies conducted in Punjab, India, and Southern Ethiopia in which the likelihood of developing stress became lower as the students advanced in their years of study [42,43]. A possible reason for this would be the self-adjustment to face the challenges encountered by the junior freshmen in terms of the new environment and academic challenges besides separation from family members and friends. The separation from pre-existing family or social support that they are familiar with, as well as the need for them to form new social interaction with students of other cultures, could also be the reason behind the higher level of mental stress among junior undergraduate students [44]. Furthermore, they also experience a high level of stress and stress-related illness as a result of heavy course workload, limited leisure time, lack of access to learning materials, and regular assessments. Comparatively, students in the higher academic levels would have adjusted better to university life by the third or fourth years. As a result, they become more mature and develop the necessary time management skills and appropriate coping strategies with daily stress [45]. Some of the intervention efforts include induction and peer mentoring programs specifically customized for first-year university students.

In addition, good sleeping quality plays a vital role in ensuring the mental wellbeing of the students to support their study process. It is also closely related to burnout [46]. The present study found that sleep quality was one of the most important factors predisposing to anxiety and depressive symptoms. Poor sleeping quality is common among university students, especially during the examination season. Many students sacrifice their sleeping hours for last-minute revisions. Nevertheless, sleep deprivation can lead to unwanted effects such as fatigue, poor cellular repair, and impaired cognitive and psychomotor functions. As a result, they suffer from poor concentration apart from having difficulty in recalling and understanding what they have studied and subsequently affecting their overall academic performances [47]. This is aggravated by the fact that those anxious students are already having difficulties falling asleep, to begin with. Similar results have been reported among university students in Ethiopia, New Zealand, and Egypt [48,49,50]. On the other hand, a meta-analysis by Baglioni et al. [51] found that non-depressive individuals with sleep problems were twice more likely to develop depression compared to those with satisfactory sleep quality. 

Headache disorders particularly migraine, tension headache, and cluster headache can also lead to a substantial disability burden. Such conditions are increasingly common among all age groups, especially the younger age groups [52]. This study highlighted a high prevalence of headaches (49.9%) among health science students. The likelihood of feeling stressed and anxious increased significantly among students who complained of a headache. Sleep issues such as fragmented sleep and insomnia may cause a headache. Nevertheless, the underlying mechanism and exact etiology remain poorly understood due to the complexity and multifactorial nature of the relationship [53]. One study reported that one in five (22%) undergraduate students suffered from headaches after consuming energy drinks to boost their study processes [54]. Additionally, other published research highlighted a high prevalence of headache (46–91%) among students taking health-related courses [52,55,56]. The frequency and severity of headaches can lead to a negative impact on the academic performance and future work performance of the students. It can also produce an unwanted socioeconomic burden on the nation given the important role of healthcare professionals in safeguarding the health of the community [57]. 

Additionally, low-grade fever is a significant risk factor for stress and anxiety in this study. A previous study by Oka et al. [58] also reported how psychological stress could produce low-grade fever (37–38 °C) in patients with chronic fatigue syndrome as well as healthy humans. They postulated that it was a result of the activation of the sympathetic nervous system that led to an increase in the core body temperature by inducing non-shivering thermogenesis in brown adipose tissue and reducing heat loss with peripheral vasoconstriction [59]. The rise in axillary and tympanic membrane temperatures, as well as a lowered finger temperature, can be associated with an increase in blood catecholamine levels. It is likely that sympathetic activation also plays an important role in temperature regulation.

In previous studies that focused on various health science programs, the students were found to have a higher-than-normal rate of mental health disorders [60,61]. Consistently, this study found different levels of SAD among different targeted academic majors. There was a significantly higher number of students majoring in Medical Imaging who were suffering from SAD compared to Medical Laboratory Sciences and Nursing students. While the exact causes remain unknown, it could be attributed to the differences in the demands, teaching methods, assessment, and grading system of each course. In the past, high levels of physical and mental occupational stressors have been reported among radiographers [62]. Unlike other health science students, Medical Imaging students received training in highly stressful computerized work environments. They also face a high workload and strict deadlines. The association between heavy computer usage and a higher prevalence of musculoskeletal and mental symptoms among undergraduate students has been reported by Reference [63]. Moreover, Medical Imaging students are also required to undergo intensive professional training that includes traditional lectures, distance learning, laboratory courses, and clinical training. Graduates from the Medical Imaging course might find that learning to cope with stress can positively affect their professional lives [64]. This might explain why Medical Imaging students’ have higher SAD levels compared with other Health Sciences students. To delineate the actual causes for this observation, future studies with a more focused investigation and different study designs are warranted.

Findings from this present study necessitate the implementation of strategies for the management of SAD in a university setting. One intervention method that could be implemented is an online standardized periodic mental health screening among university students. Previous studies conducted had shown positive outcomes in SAD management using online-based assessments [65,66]. Another strategy to manage SAD among university students is by organizing mindfulness activities. Mindfulness intervention activities that had been done online were shown to help students coping with SAD [67,68,69]. Both methods could be implemented by the university administrators for the students. 

There are certain limitations to this study; thus, caution should be practiced during the interpretation of the findings. Firstly, the cross-sectional design meant that the temporal relationship between the exposures and outcomes could not be established. Thus, future researchers should consider longitudinal studies. Next, the self-reporting mechanism in the questionnaire could have given rise to social desirability and memory recall biases that affected the outcomes. Probably this can be improved in future studies by incorporating diagnostic questionnaires to assess SAD. Thirdly, this is a single-center study in a public university in Malaysia that consists of only Bumiputra ethnic groups. There may be unique features specific to the setting and the program that influence students’ perspectives. Therefore, a multi-centered study that includes various ethnic groups would provide a better understanding of SAD among university students in Malaysia. Furthermore, we cannot conclude that gender plays a role in SAD due to the small sample size of male participants. Lastly, it is important to highlight that the DASS-21 questionnaire is only a quantitative assessment of psychological disturbance. Nevertheless, we believe that these limitations would not underestimate the significance of study results. As SAD may further progress to more serious mental health disorders, concerted efforts should be implemented to identify and prevent SAD among students. Furthermore, physical presentations such as low-grade fever, poor sleep quality, and frequent headaches that were significantly correlated with SAD were identified. These symptoms can be used by the students or academic administrators as the initial signs of mental health problems. 

## 5. Conclusions

The present study showed that the majority of Health Sciences undergraduates experienced SAD, the symptoms of mental health disorders. Anxiety was the most commonly reported among the participants, followed by depression and stress. Different risk factors were found to be associated with each condition. The findings emphasized the immediate need for healthcare professionals and university administrative personnel to provide the necessary support to this vulnerable student population. In addition, promotive efforts and counseling are needed to ensure that students would actively seek help if they are feeling stressed out. Future studies should also evaluate other factors such as the university environment (teaching-learning processes) because the psychological wellbeing of the university students does not depend solely on personal characteristics. 

## Figures and Tables

**Table 1:** Severity scores of depression, anxiety, and stress [23].

Rating	Depression	Anxiety	Stress
Normal	0–9	0–7	0–14
Mild	10–13	8–9	15–18
Moderate	14–20	10–14	19–25
Severe	21–27	15–19	26–33
Extremely Severe	28+	20+	37+

**Table 2:** Demographic characteristics of the studied participants (N = 449).

Characteristic	N (%)
Gender	
Male	69 (9.9)
Female	380 (90.1)
Ethnicity	
Malay	429 (95.5)
Others (Sabah/Sarawak)	20 (4.5)
Marital status	
Single	449 (100.0)
In a relationship/Engaged/Married	0 (0.0)
Geographical location	
Central region	113 (25.2)
Northern region	96 (21.4)
East-coast region	100 (22.3)
Southern region	93 (20.7)
Sabah/Sarawak	37 (8.2)
Did not mentioned	10 (2.2)
Year of study	
First	136 (30.3)
Second	109 (24.3)
Third	136 (30.3)
Fourth	68 (15.1)
Academic majors	
Medical Laboratory Sciences	227 (50.6)
Medical Imaging	104 (23.2)
Nursing	118 (26.2)
University CGPA	
>3.50	108 (24.1)
3.00 to 3,49	261 (58.1)
<3.00	43 (9.6)
Did not report	37 (8.2)
Education fund	
Scholarship	256 (57.0)
Self-fund	184 (41.0)
Did not report	9 (2.0)
Monthly family income	
More than RM 3000	196 (43.7)
RM 2000 to RM 2999	77 (17.1)
Less than RM 19999	159 (35.4)
Did not report	17 (3.8)
Cigarette smoking status	
Non-smoker	425 (94.7)
Current smoker	24 (5.3)

RM = Malaysian Ringgit (US$ 1 = RM 4.04 on 14 January 2021).

**Table 3:** Severity levels of depression, anxiety, and stress among the health science undergraduate students according to gender.

	Stress			Anxiety			Depression		
	Male N (%)	Female N (%)	Total N (%)	Male N (%)	Female N (%)	Total N (%)	Male N (%)	Female N (%)	Total N (%)
Normal	24 (34.8)	133 (35.0)	157 (35.0)	11 (15.9)	56 (14.7)	67 (14.9)	28 (40.6)	190 (50.0)	218 (48.6)
Mild	24 (34.8)	154 (40.5)	178 (39.6)	8 (11.6)	39 (10.3)	47 (10.5)	10 (14.5)	69 (18.2)	79 (17.6)
Moderate	15 (21.7)	73 (19.2)	88 (19.6)	14 (20.3)	114 (30.0)	128 (28.5)	20 (29.0)	82 (21.6)	102 (22.7)
Severe	4 (5.8)	16 (4.2)	20 (4.5)	12 (17.4)	89 (23.4)	101 (22.5)	8 (11.6)	28 (7.4)	36 (8.0)
Extremely severe	2 (2.9)	4 (1.1)	6 (1.3)	24 (34.8)	82 (21.6)	106 (23.6)	3 (4.3)	11 (2.9)	14 (3.1)
*p*-value (Across levels)	0.645			0.119			0.329		

**Table 4:** One-way ANOVA of the DASS-21 scores with the year of study.

Variable		DASS-21 Stress	DASS-21 Anxiety	DASS-21 Depression
Year of study	Mean Square	114.621	142.001	144.612
	F	2.940	2.576	2.065
	Sig.	0.033	0.053	0.104

**Table 5:** Analysis of variables associated with stress, anxiety and depression.

Variable		DASS-21 Stress			DASS-21 Anxiety			DASS-21 Depression		
	χ^2^	43.387, df = 11			73.607, df = 11			59.805, df = 11		
	*p*-value	<0.001			<0.001			<0.001		
	Nagelkerke R^2^	0.136			0.224			0.173		
		B	95% CI (B)	*p*-value	B	95% CI (B)	*p*-value	B	95% CI (B)	*p*-value
Gender (Female vs. Male)		0.345	0.732, 2.723	0.303	−0.290	0.481, 1.963	0.936	0.290	0.720, 2.484	0.358
Program										
Nursing (ref.)										
Medical Laboratory Sciences		0.694	1.111, 3.608	0.021 *	0.480	0.955, 2.735	0.074	0.921	1.441, 4.373	0.001 *
Medical Imaging		0.777	1.106, 4.278	0.024 *	0.821	1.053, 4.098	0.036 *	1.365	2.046, 7.361	0.001 *
Weight gain (Yes vs. No)		0.252	0.681, 2.429	0.438	0.299	0.595, 3.056	0.475	0.307	0.737, 2.507	0.326
Weight loss (Yes vs. No)		0.721	0.971, 4.357	0.060	0.632	0.603, 5.873	0.276	0.363	0.673, 3.068	0.348
Fatigue (Yes vs. No)		0.488	0.987, 2.688	0.056	0.854	1.427, 3.867	0.001 *	0.506	1.044, 2.638	0.032 *
Low-grade fever (Yes vs. No)		0.597	1.042, 3.164	0.035 *	1.072	1.242, 6.869	0.014 *	−0.162	0.484, 1.495	0.574
Smoking cigarettes (Yes vs. No)		0.313	0.503, 3.714	0.540	−0.314	0.782, 4.099	0.168	0.688	0.753, 5.258	0.165
Physical limitations (Yes vs. No)		0.452	0.862, 2.863	0.140	0.582	0.782, 4.099	0.168	−1.350	0.478, 1.599	0.662
Frequent headaches (Yes vs. No)		0.497	1.023, 2.640	0.040 ^*^	0.853	1.435, 3.839	0.001 *	0.213	0.797, 1.922	0.343
Poor sleep quality (Yes vs. No)		0.256	0.779, 2.143	0.321	0.633	1.003, 3.534	0.049 *	0.795	1.377, 3.558	0.001 *

* Significant at *p* < 0.05.

## Data Availability

The data presented in this study are available on request from the corresponding author.
